# Reviewer acknowledgement 2012

**DOI:** 10.1186/1472-6963-13-78

**Published:** 2013-04-04

**Authors:** Natalie Pafitis

**Affiliations:** 1 BioMed Central, 236 Gray's Inn Road, London, WC1X 8HB

## Abstract

**Contributing reviewers:**

The editors of BMC Health Services Research would like to thank all our reviewers who have contributed to the journal in 2012.

## 

Margunn Aanestad

Norway

Jos Aarts

Netherlands

Olaf Aasland

Norway

Penelope Abbott

Australia

Khaled Abdel-Kader

United States of America

Muhammad Ahmed Abdullah

Pakistan

Leon Adams

Australia

Kathleen Adams

United States of America

Rachael Addicott

United Kingdom

Marian Ådnanes

Norway

Bekele Afessa

United States of America

Irene Akua Agyepong

Ghana

Carolyn Ahlers-Schmidt

United States of America

Gerd Ahlstrom

Sweden

Linda Aiken

United States of America

Christopher Aisbett

Australia

Miriam Ajuba

Nigeria

Manabu Akazawa

Japan

Halida Akhter

United States of America

Emilio Alba

Spain

Michael A. Albert

Norway

Vassilis Aletras

Greece

G. Caleb Alexander

United States of America

Hisham Aljadhey

Saudi Arabia

Janet Allan

Australia

Veerasathpurush Allareddy

United States of America

Kelli Allen

United States of America

Susan Allen

United States of America

Norrina Allen

United States of America

Alex Almoudaris

United Kingdom

Yasir Alrawi

United Kingdom

Amer Alshekhlee

United States of America

Arshad Altaf

Pakistan

Osama Altayar

United States of America

Carmen Alvarez

United States of America

Sam Amirfar

United States of America

Jimoh Amzat

Nigeria

Ashwin Ananthakrishnan

United States of America

Ali Anbori

Iraq

Shilo Anders

United States of America

Rikke Sand Andersen

Denmark

Bengt Andrae

Sweden

Peter Leslie Annear

Australia

Heymann Anthony

Israel

Victoria Arango

Colombia

Yumiko Aratani

United States of America

Julian Archer

United Kingdom

Jan Arlebrink

Sweden

Gerry Armitage

United Kingdom

Judy Arnetz

United States of America

Diane Arnold-Reed

Australia

Richard Ashcroft

United Kingdom

John Ele-Ojo Ataguba

South Africa

Helen Atherton

United Kingdom

Isabelle Aujoulat

Belgium

Iben Axén

Sweden

Liat Ayalon

Israel

Syed Khurram Azmat

Pakistan

Stella Babalola

United States of America

Tobias Back

Germany

Motasim H.Y Badri

South Africa

Jong-Deuk Baek

United States of America

Kristine Bærøe

Norway

Juan Baeza

United Kingdom

Julia Bailey

United Kingdom

Ross Bailie

Australia

Lesley Bainbridge

Canada

Solome Kiribakka Bakeera

Uganda

Rachel Baker

United Kingdom

Amanda Baker

Australia

Danial Baker

United States of America

Ileana Baldi

Italy

Stefano Balducci

Italy

Jane Ball

United Kingdom

Joyce Balls-Berry

United States of America

Jane Banaszak-Holl

United States of America

Emily Banks

Australia

Raquel Barba

Spain

Anna Barker

Australia

Amber Barnato

United States of America

Daniel Barnett

United States of America

Adrian Barnett

Australia

Sandro Barni

Italy

Joaquin Barnoya

Guatemala

Giovanni Barosi

Italy

Judith Barr

United States of America

David Barron

United Kingdom

Sabine Bartholomeyczik

Germany

John Bartlett

United States of America

Mathias Basner

United States of America

Richard Alan Batley

United Kingdom

John Batsis

United States of America

Catherine Battaglia

United States of America

Marie-Dominique Beaulieu

Canada

Eddy Beck

Switzerland

Davida Becker

United States of America

Helene Bednarsh

United States of America

Rene Bekker

Netherlands

Nathaniel Bell

Canada

Ajediran Bello

Ghana

Jim Bellows

United States of America

Abdulbari Bener

Qatar

Ramona Benkert

United States of America

Ria Benko

Hungary

Miguel Bennasar-Veny

Spain

David Ben-Tovim

Australia

John E. Berg

Norway

Anne-Marie Bergh

South Africa

Raoul III Bermejo

Philippines

Larry H Bernstein

United States of America

Stan Bernstein

United States of America

Andrés Berruti

United States of America

Heiner K. Berthold

Germany

Jan Beyersmann

Germany

Padma Bhate-Deosthali

India

Sohinee Bhattacharya

United Kingdom

Onil Bhattacharyya

Canada

Graham Bickler

United Kingdom

Regien Biesma

Ireland

Silvia Bigatti

United States of America

Nikos Bilanakis

Greece

Claudio Bilotta

Italy

Annette Bishop

United Kingdom

Roger Bivand

Norway

Anne Mette Bjerkan

Norway

Oyvind Bjertnaes

Norway

Amanda Blackford

United States of America

Karl Blanchet

United Kingdom

Andrew Blann

United Kingdom

Nicole Blay

Australia

Mary Blegen

United States of America

Alison Blenkinsopp

United Kingdom

Ilse Blignault

Australia

Christoph Bobrowski

Germany

Said Bodur

Turkey

Andreas Böhmer

Germany

Barbara Bokhour

United States of America

Mary Bollinger

United States of America

Billie Bonevski

Australia

Judith Booij

Netherlands

Heather Boon

Canada

Camille Boostrom

Ireland

Luisa Borrell

United States of America

Laurent Boyer

France

Marek Brabec

Czech Republic

Eleanor Bradley

United Kingdom

Heather Bradley

United States of America

Fay Bradley

United Kingdom

Elizabeth Bradley

United States of America

Heather Bradley

United States of America

Søren Brage

Norway

Heather Brandt

United States of America

Peter Brawer

United States of America

Peter Brieger

Germany

William Brieger

United States of America

Melissa Brouwers

Canada

Stephanie Brown

Australia

Roger Brown

United States of America

Adalsteinn Brown

Canada

Tim Bruckner

United States of America

Brian Bruen

United States of America

Julie Bruneau

Canada

Elisa Bruno

Italy

Carlo Bruno

Italy

Stirling Bryan

Canada

Wendy Bryant

United Kingdom

Petra Brzank

Germany

Daniel Budnitz

United States of America

Awang Bulgiba

Malaysia

Monika Bullinger

Germany

Ersilia Buonomo

Italy

Alex Burdorf

Netherlands

Frederick Burge

Canada

Patrick Burke

United States of America

Olivier Busch

Netherlands

Charles Butts

Canada

Penny Buykx

Australia

Peter Byass

Sweden

Jens Byskov

Denmark

Dominique Anne-Michele Cadilhac

Australia

Carol Cain

United States of America

Stefano Calciolari

Switzerland

Raff Calitri

United Kingdom

Chantal Camden

Canada

Donatella Camerino

Italy

Ian Cameron

Australia

Linda Cameron

United States of America

Christine Campbell

United Kingdom

John Campbell

United Kingdom

Daniel Campeau

Canada

Marylou Cardenas-Turanzas

United States of America

Mariko Carey

Australia

Benedicte Carlsen

Norway

Christopher Carroll

United Kingdom

Jenny Carryer

New Zealand

Dean Carson

Australia

Ewart Carson

United Kingdom

Bendix Carstensen

Denmark

Silvana Castaldi

Italy

Ken Catchpole

United States of America

Gillian Caughey

Australia

Aaron Caughey

United States of America

Thomas Cecil

United Kingdom

Carlos Centeno

Spain

Nzapfurundi Chabikuli

Nigeria

Nirali Chakraborty

United States of America

John Chalker

United States of America

Kalipso Chalkidou

France

Bibiana Chan

Australia

Hung-Hao Chang

Taiwan

Simon Chang

China

Sarika Chaturvedi

India

Allen Cheadle

United States of America

Brian Chen

United States of America

Guoqing Chen

United States of America

Zhuo Chen

United States of America

Jack Chen

Australia

Qi Chen

United States of America

Guanmin Chen

Canada

Yingyao Chen

China

Yu-Chun Chen

Taiwan

Liang-Kung Chen

Taiwan

Zhuo Chen

United States of America

Yu-Ting Cheng

Taiwan

Benjamin Chesluk

United States of America

Ivor Chestnutt

United Kingdom

Daniel Ching

United Kingdom

Catherine Chittleborough

United Kingdom

Catherine Chittleborough

Australia

Ching-Ju Chiu

Taiwan

Gabriel Chodick

Israel

Lumbwe Chola

South Africa

Maria Chorozoglou

United Kingdom

Yiing-Jenq Chou

Taiwan

Mahbub Chowdhury

Bangladesh

Tom Christensen

Norway

Lisa Bøge Christensen

Denmark

Terkel Christiansen

Denmark

Siew Siang Chua

Malaysia

Ogoamaka Ifeagachukwu Chukwuogo

Nigeria

Kuo-Piao Chung

Taiwan

Kathryn Church

United Kingdom

Neree Claes

Belgium

Richard Clancy

Australia

Fiona Clement

Canada

Christopher John Clements

Australia

Virginie Cobigo

United Kingdom

Michael Coffey

United Kingdom

Simon Cohn

United Kingdom

Dirk Colaert

Belgium

Preciosa Coloma

Netherlands

Ann Colosia

United States of America

DeLawnia Comer-HaGans

United States of America

Elizabeth Comino

Australia

Amelia Compagni

Italy

Mark Connolly

Switzerland

Kay Cooper

United Kingdom

Paula Cooper

United Kingdom

Nicola Cooper

United Kingdom

Jeremy Coquart

France

Martine Corijn

Belgium

Ignacio Correa-Velez

Australia

William Corser

United States of America

Aline Corvol

Germany

Andrew Costa

Canada

Massimo Costantini

Italy

Jonathan Cox

United Kingdom

Peter Cram

United States of America

Jane Murray Cramm

Netherlands

Peter Crampton

New Zealand

Sarah Crane

United States of America

Bart Criel

Belgium

Frances Cunningham

Australia

Vernon Curran

Canada

Janet Curran

Canada

Douglas Curran-Everett

United States of America

Craig J. Currie

United Kingdom

Kay Currie

United Kingdom

David Currow

Australia

Lesley Curtis

United States of America

Andrea Curtis

Australia

Simon Dagenais

United States of America

Synneve Dahlin-Ivanoff

Sweden

Helen Dakin

United Kingdom

Sarah Damery

United Kingdom

Gianfranco Damiani

Italy

Lalit Dandona

India

Karen Daniels

South Africa

Christian Darras

Belgium

Mahua Das

United Kingdom

Abhijit Das

India

Shamita Das Dasgupta

United States of America

Amy Davidoff

United States of America

Elizabeth Davies

United Kingdom

Sheryl Davies

United States of America

Manuela De Allegri

Germany

Cees de Baat

Netherlands

Dinny de Bakker

Netherlands

Geertruida De Bock

Netherlands

Beatriz de Camargo

Brazil

Frank de Charro

Netherlands

Ayesha De Costa

Sweden

Pierpaolo De Feo

Italy

Tony De Groote

Belgium

Satiro De Oliveira

United States of America

Frank De Paepe

Belgium

Matthieu de Stampa

France

Katinka de Wet

South Africa

Trudi Deakin

United Kingdom

Eugene Declercq

United States of America

Howard Degenholtz

United States of America

Narayana Delampady

India

Eren Demir

United Kingdom

Sarah Dennis

Australia

Sophie Derchain

Brazil

Sarah Derrett

New Zealand

Annabel Desgrees-du-Lou

France

Suely Deslandes

Brazil

Walter Devillé

Netherlands

Franklin Dexter

United States of America

Paola Di Carlo

Italy

Alba DiCenso

Canada

Marjolein Dieleman

Netherlands

Javier Diez-Domingo

Spain

Michelle DiGiacomo

Australia

Tinne Dilles

Belgium

Nafees Din

United Kingdom

Yan Ding

Germany

Yan Ding

Germany

Francois Dionne

Canada

Simon Dixon

United Kingdom

Lisa Dixon

United States of America

Mai Do

United States of America

Charles Doarn

United States of America

Chris Doecke

Australia

James Doidge

Australia

Lisa Dolovich

Canada

Hengjin Dong

China

Amol Dongre

India

Tim Doran

United Kingdom

Pamela Doty

United States of America

William Doucette

United States of America

Abdel Douiri

United Kingdom

Karla Douw

Denmark

David Dowdy

United States of America

Amy Downing

United Kingdom

Michelle Dowsey

Australia

Alizon Draper

United Kingdom

Irena Draskovic

Netherlands

Jennifer Duchon

United States of America

Martin Duerden

United Kingdom

Paul Dugdale

Australia

Aaron Dumont

United States of America

Eilidh Duncan

United Kingdom

David Dunt

Australia

Luis Durán-Arenas

Mexico

Gilles Dussault

Portugal

Richard Edlin

New Zealand

John Edmonstone

United Kingdom

Kristina Edvardsson

Sweden

Sandra Edwards

United States of America

Adrian Edwards

United Kingdom

Phil Edwards

United Kingdom

Stephan Ehrhardt

United States of America

Arne H Eide

Norway

Kristjana Einarsdottir

Australia

Jonathan Einbinder

United States of America

Eva Ekvall Hansson

Sweden

Keith Elder

United States of America

Shareen El-Ibiary

United States of America

Fadi El-Jardali

Lebanon

Tarig Elraiyah

United States of America

Abdulrahman El-Sayed

United States of America

Jon Emery

Australia

Martin Emmert

Germany

Jane Chinelo Enemuoh

Nigeria

Mike English

Kenya

Rajiv Erasmus

South Africa

Nicole Ernstmann

Germany

Jason Etchegaray

United States of America

Arash Etemadi

Iran

Enyi Etiaba

Nigeria

Remare Ettarh

Kenya

Meirion Evans

United Kingdom

Christine Everett

United States of America

Soludo Eze

Nigeria

Ogochukwu Ezeoke

Nigeria

Nkoli Ezumah

Nigeria

Marjan Faber

Netherlands

Wen-Feng Fang

Taiwan

Cheng-Chung Fang

Taiwan

Valeria Fano

Italy

Andrew Farmer

United Kingdom

Saeed Farooq

Pakistan

Saeed Farooq

United Kingdom

Zafar Fatmi

Pakistan

Thomas Faunce

Australia

Annunziata Faustini

Italy

Stefan Felder

Germany

Xing Lin Feng

China

Judith Fethney

Australia

Alexander Fiks

United States of America

Patricia Findley

United States of America

Kathleen Finlayson

Australia

Patrik Finne

Finland

Michael Fischer

United States of America

Jacqueline Fitzgerald

United Kingdom

Hans Flaatten

Norway

Richard Fleming

Australia

Steffen Flessa

Germany

Marie-Josée Fleury

Canada

Signe Flottorp

Norway

Francesca Foltran

Italy

Lindsay Forbes

United Kingdom

John Ford

United Kingdom

Jay Ford

United States of America

Roberto Forero

Australia

Leena Forma

Finland

Karina Forrest Perkins

United States of America

Birger Carl Forsberg

Sweden

Yvonne Forsell

Sweden

Marianna Fotaki

United Kingdom

Lynne Franco

United States of America

Linda Rose Frank

United States of America

Bryony Dean Franklin

United Kingdom

Martin Friedberg

Canada

Roland Friele

Netherlands

Christopher Friese

United States of America

Karen Frith

United States of America

Nina Fudge

United Kingdom

Tomomi Fujisawa

Japan

Jeffrey Fuller

Australia

Lawrence Fulton

United States of America

Constance Fung

United States of America

John Furler

Australia

Carl Johan Fürst

Sweden

Danilo Fusco

Italy

Sander Gaal

Netherlands

Anna Gagliardi

Canada

Mariana Galante

Argentina

Jenny Gallagher

United Kingdom

Adeline Gallini

France

Livio Garattini

Italy

Montse Garcia

Spain

Sara Garfield

United Kingdom

Paul Garner

United Kingdom

Andrew Garratt

Norway

Robin Gauld

New Zealand

Angèle Gayet-Ageron

Switzerland

Benjamin Geisler

Germany

Joan Gene Badia

Spain

Andrew Georgiou

Australia

Andreas Gerber

Germany

Christian Gericke

United Kingdom

Oke Gerke

Denmark

Robyn Gershon

United States of America

Paul Giesen

Netherlands

Ana Gil Lacruz

Spain

Luis Andrés Gimeno-Feliu

Spain

Paolo Giorgi Rossi

Italy

Mika Gissler

Finland

Isabella Laura Giusepi

Italy

Beryl Primrose Gladstone

Germany

Richard Glazier

Canada

Brian Godman

Sweden

Katja Goetz

Germany

Isabel Goicolea

Sweden

Rachel Gold

United States of America

Michael Goldacre

United Kingdom

Neil Goldfarb

United States of America

Lauren Goldman

United States of America

Daniela Goncalves

United Kingdom

Steve Goodacre

United Kingdom

Claire Goodman

United Kingdom

Eric Goplerud

United States of America

Zulfikar Gorar

Pakistan

Louisa Gordon

Australia

Allan Goroll

United States of America

Holger Gothe

Austria

Christopher Graber

United States of America

Sherry Grace

Canada

Peter Granger

Canada

Darryl Gray

United States of America

Felix Greaves

United Kingdom

Beverly Green

United States of America

Ellen Green

United States of America

Carla A. Green

United States of America

Colin Green

United Kingdom

William Greene

United States of America

Jessica Greene

United States of America

Geva Greenfield

Israel

Trisha Greenhalgh

United Kingdom

Susan Griffin

United Kingdom

John R Griffith

United States of America

Oliver Groene

Spain

Oliver Groene

United Kingdom

Peter Groenewegen

Netherlands

Erik Groessl

United States of America

Vigdis Abrahamsen Grøndahl

Norway

Catharina Groothuis-Oudshoorn

Netherlands

Scott Grosse

United States of America

Axel Grothey

United States of America

DaShawn Groves

United States of America

Andrea Gruneir

Canada

Gary Grunwald

United States of America

Danan Gu

United States of America

Francisco Gude

Spain

P. Cristian Gugiu

United States of America

Laszlo Gulacsi

Hungary

Janice Gullick

Australia

Jeff Guo

United States of America

Mary Gurney

United States of America

Ipek Gurol-Urganci

United Kingdom

Rikke Gut

Denmark

Jillian Guthrie

Australia

Iñaki Gutierrez

Spain

Joseph Guydish

United States of America

Stella Gwini

Australia

Dorte Gyrd-Hansen

Denmark

Marion Haas

Australia

Karen Hacker

United States of America

Azar Hadadi

Iran

Emina Hadziabdic

Sweden

Jeannie Haggerty

Canada

Amy Hagopian

United States of America

Hossein Haji Ali Afzali

Australia

Katja Marja Hakkarainen

Sweden

Sharon Hakkennes

Australia

Elizabeth Halcomb

Australia

Patricia Halfon

Switzerland

Carmen Hall

United States of America

Alison Hamilton

United States of America

Antje Hammer

Germany

Theodore Hammett

United States of America

Euna Han

Korea, South

Christine Hancock

United States of America

Stephen Robert Hanney

United Kingdom

Ben Hannigan

United Kingdom

Juha Hänninen

Finland

Claudia Hanson

Sweden

Yanhua Hao

China

Anita Hardon

Netherlands

Elaine Hargreaves

New Zealand

Karen Harlos

Canada

John Harrison

United Kingdom

Janneke Harting

Netherlands

Syed Shahrukh Hashmi

United States of America

Syed Farid-ul- Hasnain

Pakistan

Mohamed Azmi Hassali

Malaysia

Karen Hassell

United Kingdom

Daniël Haverkamp

Netherlands

Les Hayduk

Canada

Laureen Hayes

Canada

Mark Hayward

United Kingdom

Vicky Head

United Kingdom

Judith Healy

Australia

Larry Hearld

United States of America

Emma Heeley

Australia

Ulrike Held

Switzerland

Susanne Hempel

United States of America

Michelle Hendriks

Netherlands

Joan Henriksen Hellyer

United States of America

Lori Hensic

United States of America

Michael Herdman

Spain

Katja Hermann

Germany

Carlos Hernandez Giron

Mexico

Melba Hernandez-Tejada

United States of America

Annemie Heselmans

Belgium

Jeanne Hewitt

United States of America

Mary Hickson

United Kingdom

Isabel Higgins

Australia

Irene J Higginson

United Kingdom

Peter Hill

Australia

Andrew Hill

New Zealand

Andrea Hilton

United Kingdom

David Himmelstein

United States of America

Susan Hindle-Smith

United Kingdom

Bradford Hirsch

United States of America

Katarina Hjelm

Sweden

Peter Hjertholm

Denmark

Michael Ho

United States of America

Jason Hockenberry

United States of America

Falk Hoffmann

Germany

Barbara Hoffmann

Germany

Dag Hofoss

Norway

Christa Hojlo

United States of America

Diane Holland

United States of America

Jennifer Hollowell

United Kingdom

Margaret Holmes-Rovner

United States of America

Charles Hongoro

South Africa

Roderick Hooker

United States of America

Michael Horberg

United States of America

Patrick Horner

United Kingdom

Munier Hossain

United Kingdom

Einar Hovlid

Norway

Krista Howard

United States of America

Rachel Howard

United Kingdom

Chi-yuan Hsu

United States of America

Ya-Seng (Arthur) Hsueh

Australia

Guoqing Hu

China

Teh Wei Hu

United States of America

Xuan-Yi Huang

Taiwan

Wei Huang

United States of America

Yu-Ying Huang

Taiwan

Yu-tung A. Huang

Taiwan

Nicole Huang

Taiwan

Peter Hudson

Australia

Claudia Huebner

Germany

José María Huerta

Spain

Ronda Hughes

United States of America

Robbert Huijsman

Netherlands

Cynthia Hunter

Australia

Jeremiah Hurley

Canada

Deirdre Hurley

Ireland

Nusrat Husain

United Kingdom

Sally Huston

United States of America

David Hutton

United States of America

Wei-Ting Hwang

United States of America

Norman Hymowitz

United States of America

Sylvia Hysong

United States of America

Esther Iecovich

Israel

Yuichi Imanaka

Japan

Michelle Irving

Australia

Hilde Hestad Iversen

Norway

Hilde Hestad Iversen

Norway

Veena Iyer

India

Susan Jack

Canada

Jeanette Jackson

United Kingdom

Sally Jacobs

United Kingdom

Bart Jacobs

Laos

Delyth James

United Kingdom

Janko Jankovic

Serbia

Dominic Janssen

United Kingdom

Barbara Janssen-Solberg

Netherlands

Rich Janzen

Canada

Sara Javanparast

Australia

Hiranthi Jayaweera

United Kingdom

Jalila Jbilou

Canada

Wen-Yuan Jen

Taiwan

Paul Jennings

Australia

Natasja Koitzsch Jensen

Denmark

Yun-Hee Jeon

Australia

Anthony Jerant

United States of America

Rebecca Jester

United Kingdom

Huanguang Jia

United States of America

Moyez Jiwa

Australia

Emmanuel John

United States of America

Helen Johnson

Australia

Peter Johnson

United States of America

Sonia Johnson

United Kingdom

Vanessa Johnston

Australia

Bridget Johnston

United Kingdom

Kerina Jones

United Kingdom

Alan Jones

United States of America

Emily Jones

United States of America

Wu Joseph

Hong Kong

Rohina Joshi

Australia

Sue Jowett

United Kingdom

Tanisha Jowsey

Australia

Andrew Judge

United Kingdom

Kenneth Judge

United Kingdom

Angela Jukkala

United States of America

Minsoo Jung

United States of America

Pirjo Juvonen-Posti

Finland

Jeremy Kahn

United States of America

Leila Kahwati

United States of America

Klaus Kaier

Germany

László Kalabay

Hungary

John Kalbfleisch

United States of America

Karin Källander

Uganda

Elizabeth Kalucy

Australia

Sumit Kane

Netherlands

Barun Kanjilal

India

Lydia Kapiriri

Canada

Ajay Kapur

United States of America

Fatma Karapinar

Netherlands

Jon Karnon

Australia

Chandima Karunanayake

Canada

Moses Katabarwa

United States of America

Hugo Katus

Germany

Jeffrey Katz

United States of America

Lewis Kazis

United States of America

Abby Kazley

United States of America

Sheila Keane

Australia

Guy Kegels

Belgium

Margaret Kelaher

Australia

Helen Keleher

Australia

Daniel Kelly

United Kingdom

Alex Kemper

United States of America

Sally Kendall

United Kingdom

Caitlin Kennedy

United States of America

Elissa Kennedy

Australia

Anne Kennedy

United Kingdom

Stephen Kerr

Australia

Ngaire Kerse

New Zealand

Kiarri Kershaw

United States of America

Laila Khalid

United States of America

Hossein Khalili

Iran

M Mahmud Khan

United States of America

Murad Khan

Pakistan

Nada Khan

United Kingdom

Rahul Khanna

United States of America

Nandita Khera

United States of America

Ghafur Khodjamurodov

Tajikistan

Liaquat Khowaja

Pakistan

Arif Khwaja

United Kingdom

Andrew Kidd

United Kingdom

Karina Kielmann

United Kingdom

Amy Kilbourne

United States of America

Reinhold Kilian

Germany

Helen Killaspy

United Kingdom

Sue Kilpatrick

Australia

Seo Young Kim

United States of America

Dae Hyun Kim

United States of America

In Kyu Kim

United States of America

Scott Kim

United States of America

Joyce Kinaro

Kenya

Karen Kinder

Germany

John Kinsman

Sweden

Sue Kirby

Australia

Marit Kirkevold

Norway

Marilyn Kirshbaum

United Kingdom

Lars Kjellin

Sweden

Ruth M. Kleinpell

United States of America

Marie Klingberg-Allvin

Sweden

Felicia Knaul

United States of America

John Knight

Canada

Sabina Knight

Australia

Jeroen Knipscheer

Netherlands

Birthe Loa Knizek

Norway

Vikki Knott

Australia

Hannah Knudsen

United States of America

Merril Knudtson

Canada

Gülseren Kocaman

Turkey

Tracey Perez Koehlmoos

Bangladesh

Stephen Kogut

United States of America

Friedrich Köhler

Germany

Simo Kokko

Finland

Niina Kolehmainen

United Kingdom

Masahide Kondo

Japan

Karna Georges Kone

Canada

Ayumi Kono

Japan

Evangelos Kontopantelis

United Kingdom

Deborah Kopansky-Giles

Canada

Meera Kotagal

United States of America

Anita Kotwani

India

Gab Kovacs

Australia

Gunilla Krantz

Sweden

Allan Krasnik

Denmark

Sara Kreindler

Canada

Solvejg Kristensen

Denmark

Jimmie Kristensson

Sweden

Maarten Krol

Netherlands

Thilo Kroll

United Kingdom

Margaret Elizabeth Kruk

United States of America

Elizabeth Krupinski

United States of America

Leighton Ku

United States of America

Tomohide Kubo

Japan

Suzan Kucukarslan

United States of America

Ellen Kuhlmann

Germany

Ernst Kuipers

Netherlands

Jennifer Kuk

Canada

Judith Kulig

Canada

Rashmi Kumar

India

Rajesh Kumar

India

Ramesh Kumar

Pakistan

Kari Kvaerner

Norway

Chun Shing Kwok

United Kingdom

Soonman Kwon

Korea, South

Mylene Lagarde

United Kingdom

Ronald Lagoe

United States of America

Richard Laing

Switzerland

Jeroen Lakerveld

Netherlands

Christiaan Lako

Netherlands

Sean Lam

Singapore

Marie-Laurence Lambert

Belgium

Veronica Lambert

Ireland

Anthony Daniel LaMontagne

Australia

Astrid Langer

Germany

Malinee Laopaiboon

Thailand

Allison Larg

Australia

Matthew Large

Australia

Heather Laschinger

Canada

Fatimah Lateef

Singapore

Denys Lau

United States of America

Francis Lau

Canada

Tessa Laubscher

Canada

Carl Lavie

United States of America

Christina Lazar

United States of America

Eduardo Lazcano-Ponce

Mexico

Frederic Le Marcis

France

Camille Le Ray Le Ray

France

Maria Lucia Lebrao

Brazil

Chang Won Lee

Korea, South

Susan Lee

Australia

Mandy Lee

Ireland

Yue-Chune Lee

Taiwan

Sook-Jeong Lee

Korea, South

Wui-Chiang Lee

Taiwan

Joy Lee

United States of America

France Legare

Canada

David Legge

Australia

Juhani Lehto

Finland

Hanna Leicht

Germany

Margus Lember

Estonia

Heidi Lempp

United Kingdom

Eugene Lengerich

United States of America

Emmanuel Lesaffre

Belgium

Pierre Levy

France

Charlotte Lewis

United States of America

Joel Lexchin

Canada

Luci Leykum

United States of America

Ian Li

Australia

Guohong Li

China

Nicola Lucio Liberato

Italy

Sharon Licqurish

Australia

Tzeng Jih Lin

Taiwan

Swu-Jane Lin

United States of America

Xu Lin

China

Wender Lin

Taiwan

Ming-Hwai Lin

Taiwan

Marianne L Lindahl

Sweden

Lars Lindholm

Sweden

Richard Lindley

Australia

Lee Lindquist

United States of America

Amy Linsky

United States of America

Christos Lionis

Greece

Wendy Lipworth

Australia

Peter Littlejohns

United Kingdom

Chuan-Fen Liu

United States of America

George Liu

Australia

Zhaorui Liu

China

Li Liu

United States of America

Xiaoyun Liu

China

Rickard Ljung

Sweden

Jocelyn Lockyer

Canada

Carl Lombard

South Africa

Emilia Lombardi

United States of America

Nazir Iftikhar Lone

United Kingdom

Sergio Lopes

Brazil

Stephen Lord

Australia

Paula Lorgelly

Australia

Carlo Lucioni

Italy

Ramon Luengo-Fernandez

United Kingdom

Isaac Luginaah

Canada

Pisake Lumbiganon

Thailand

Ithai Lurie

United States of America

Brigid Lynch

Australia

Cheryl Lynch

United States of America

David Lyon

United Kingdom

Georgios Lyratzopoulos

United Kingdom

Hans Maarse

Netherlands

Jane Macha

Tanzania

Alessandra Maciel Almeida

Brazil

Michael Maciosek

United States of America

Peter MacPherson

Malawi

Peggy J. Maddox

United States of America

Thomas Maddox

United States of America

Shoichi Maeda

Japan

Parker Magin

Australia

Robert Magnani

Indonesia

Gayenell Magwood

United States of America

Rajendra Maharaj

South Africa

Qun Mai

Australia

Ian Maidment

United Kingdom

Michele Maiers

United States of America

Frances Mair

United Kingdom

Shaheen Majid

Singapore

Peter Makai

Netherlands

Samir Malhotra

India

Sunil Raja Manandhar

Nepal

Roshni Mangalore

United Kingdom

Maria Piera Mano

Italy

Jonathan Mant

United Kingdom

Renato Mantegazza

Italy

Jill Manthorpe

United Kingdom

Vittorio Mapelli

Italy

Siana Mapunjo

Tanzania

Perla Marang-van de Mheen

Netherlands

Bruno Marchal

Belgium

Ludmila Marcinowicz

Poland

Closon Marie-Christine

Belgium

Amelia Marques

Brazil

Suzanne Martin

United Kingdom

Amy Martin

United States of America

Greg Martin

New Zealand

Bradley Martin

United States of America

Iñaki Martin Lesende

Spain

Adolfo Martinez Valle

Mexico

Melinda Martin-Khan

Australia

Carmen Martos

Spain

Grant Martsolf

United States of America

Daniele Mascia

Italy

Helen Mason

United Kingdom

Siriel Massawe

Tanzania

Tsitsi Masvawure

Uruguay

Kedar Mate

United States of America

Ceu Mateus

Portugal

Catherine Mathews

South Africa

Nancy Matthew-Maich

Canada

Carl May

United Kingdom

Arslan Mazhar

Pakistan

Sumit Mazumdar

India

Chinyere O Mbachu

Nigeria

Godfrey Michael Mbaruku

Tanzania

Leonard Mboera

Tanzania

Anthony Mbonye

Uganda

Ryan McAllister

Australia

John McCarthy

United States of America

Melissa McCarthy

United States of America

Candace McClure

United States of America

Alex McConnachie

United Kingdom

Bryan McCormick

United States of America

Neil McDevitt

United States of America

Andrew McDonagh

United Kingdom

Geoff McDonnell

Australia

Elizabeth McInnes

Australia

Edwin David McIntosh

United Kingdom

Emma McIntosh

United Kingdom

Meredith McIntyre

Australia

Diane McIntyre

South Africa

Ellen McIntyre

Australia

Kirsten McKenzie

Australia

Ann McKillop

New Zealand

Andrew McLachlan

Australia

Steven McPhail

Australia

Deborah McWilliams

United States of America

Patrick D. Meek

United States of America

Gul Rukh Mehboob

Pakistan

Ateev Mehrotra

United States of America

Juan Manuel Mejia-Arangure

Mexico

Matthew Menear

Canada

Heather Menne

United States of America

Devidas Menon

Canada

Catherine Mercer

United Kingdom

Mario Merialdi

Switzerland

Ryan Merkow

United States of America

Nadine Meskens

Belgium

Herman Meulemans

Belgium

Thorsten Meyer

Germany

Patrik Midlöv

Sweden

Barbara Mildon

Canada

Ted Miller

United States of America

William Miller

United States of America

Katherine Mills

United States of America

Ruairidh Milne

United Kingdom

Milan Milosevic

Croatia

Jonathan Minton

United Kingdom

Motlatso Gladys Mlambo

South Africa

Kaelan Moat

Canada

Ateesha Mohamed

United States of America

Sandra Moll

Canada

Muthu Rama Krishnan Mookiah

Singapore

Vincent Mor

United States of America

Sudhakar Morankar

Ethiopia

Michel Moreau

Belgium

Myfanwy Morgan

United Kingdom

Roland Morley

United Kingdom

Elaine Morrato

United States of America

Joanna Morrison

Nepal

Richard Morriss

United Kingdom

Rachael Morton

Australia

Francesco Moscone

United Kingdom

David Moskowitz

United States of America

Henry Mosley

United States of America

Mathieu Moslonka-Lefebvre

France

Lawrence Moulton

United States of America

Gail Anne Mountain

United Kingdom

David Mountain

Australia

Vladimir Mouraviev

United States of America

Lorna Moxham

Australia

Gemini Joseph Mtei

Tanzania

Miranda Mugford

United Kingdom

Laura Muldoon

Canada

Judy Mullan

Australia

Dirk Müller

Germany

Andrew Murphy

Ireland

Carolyn Murray

Australia

Elizabeth Murray

United Kingdom

Ellen Murray

United Kingdom

Nanette Mutrie

United Kingdom

Germano Mwabu

Kenya

Aziza Joseph Mwisongo

Tanzania

Puja Myles

United Kingdom

Harriet Nabudere

Uganda

Farooq Naeem

Pakistan

Nisha Naicker

South Africa

Miharu Nakanishi

Japan

Jolly Nankunda

Uganda

Jose Luis Navarro-Espigares

Spain

Adrianne Nelson

United States of America

Ann Netten

United Kingdom

Josh Neumiller

United States of America

Peter Newcombe

Australia

Mattias Neyt

Belgium

David Ng

Canada

Chiu-Wan Ng

Malaysia

Tu Ngo

United States of America

Anh Ngo

Viet Nam

Surachat Ngorsuraches

Thailand

Nam Nguyen

Viet Nam

Anthony Nguyen

Australia

Anna Nieboer

Netherlands

Jan G. Nijhuis

Netherlands

Evalill Nilsson

Sweden

Akihiro Nishi

United States of America

Sania Nishtar

Pakistan

Barnabas Njozing

Kenya

Lungiswa Nkonki

South Africa

Mirko Noordegraaf

Netherlands

Carl Erik Nord

Sweden

Mette Nørgaard

Denmark

Richard Norman

Australia

Susan L Norris

United States of America

Rachel North

United Kingdom

Matthias Nuebling

Germany

Sabina Nuti

Italy

Achunam Nwabueze

Nigeria

Lennarth Nyström

Sweden

Ikechukwu Obi

Nigeria

Celestino Obua

Uganda

Patrick O'Connor

United States of America

Laura Odell

United States of America

Owen O'Donnell

Greece

Kate O'Donnell

United Kingdom

Thomas O'Dowd

Ireland

Uchenna Ofoma

United States of America

Cath O'Halloran

United Kingdom

Daniel Okenu

United States of America

Okore Okorafor

South Africa

Ijeoma Okoronkwo

Nigeria

Tuoyo Okorosobo

Switzerland

Kari Olson

United States of America

Bolajoko O. Olusanya

Nigeria

Heather O'Mahen

United Kingdom

Mei-Sing Ong

Australia

Chima Onoka

Nigeria

Obinna Onwujekwe

Nigeria

Wija Oortwijn

Netherlands

Christopher Orach

Uganda

Claire O'Reilly

Australia

Jackson Orem

Uganda

Titilayo O Oshodi

United Kingdom

Petr Ostadal

Czech Republic

Doris Østergaard

Denmark

Jan Ostermann

United States of America

Angel Otero

Spain

Kevin O'Toole

Australia

Lixin Ou

Australia

Jurriaan Oudhoff

United Kingdom

David Owen

United Kingdom

Jorma Paavonen

Finland

Fred Paccaud

Switzerland

Katie Page

Australia

Punam Pahwa

Canada

Simon Palfreyman

United Kingdom

Ståle Pallesen

Norway

Walter Palmas

United States of America

Jaideep Pandit

United Kingdom

Massimiliano Panella

Italy

Peter Pang

United States of America

George Pariyo

Switzerland

Melissa Parker

United States of America

Roger Parslow

United Kingdom

Helen Parsons

United States of America

H. Roeline W. Pasman

Netherlands

Daksha Patel

United Kingdom

Brijesh Patel

United Kingdom

Julietta Patnick

United Kingdom

Calum Paton

United Kingdom

Toby Pavey

United Kingdom

Heather Payne

United Kingdom

Sheila Payne

United Kingdom

Jim Pearse

Australia

David Pearson

United States of America

Geir Pedersen

Norway

Salvador Peiró

Spain

Roni Peleg

Israel

Zongchao Peng

China

Julian Perelman

Portugal

Ricardo Pérez-Núñez

Mexico

David Perkins

Australia

Shoa-Jen Perng

Taiwan

Brian Perron

United States of America

Tania Perrone

Italy

Stephen Persell

United States of America

John Petkov

Australia

Konstantinos Petsios

Greece

Max Petzold

Sweden

Mai Pham

Australia

Anastas Philalithis

Greece

Mit Philips

Belgium

Christine Phillips

Australia

Giulio Pioli

Italy

Marie Pirotta

Australia

Magali Pirson

Belgium

Maurice Place

United Kingdom

Elena Platonova

United States of America

Jenny Ploeg

Canada

Jennifer Pohlhaus

United States of America

Subhash Pokhrel

United Kingdom

Claudio Politi

Switzerland

Riccardo Polosa

Italy

Marie-Pascale Pomey

Canada

John DH Porter

United Kingdom

Shirley Porterfield

United States of America

Gawaine Powell Davies

Australia

Birte Prahl-Andersen

Netherlands

Rebekah Pratt

United States of America

Anna Prenestini

Italy

Valerie Press

United States of America

Justin Presseau

United Kingdom

Jennifer Price

Canada

Jochen Profit

United States of America

Paul Pronyk

United States of America

Denis Protti

Canada

Keith Provan

United States of America

Christy Pu

Taiwan

Jacqueline Pugh

United States of America

Enriqueta Pujol-Ribera

Spain

Sarah Purdy

United Kingdom

Bernd Puschner

Germany

Juha Puustjärvi

Finland

Jamal Qaddumi

Palestinian Territory

Harith Qazaz

Iraq

Dongfu Qian

China

Cameron Quanbeck

United States of America

Wilm Quentin

Germany

Denise Quigley

United States of America

Mohammad Rab

Canada

Jean-Sebastien Rachoin

United States of America

Kurupath Radhakrishnan

India

Arona Ragins

United States of America

Sanjay Rai

India

Shawn Ralston

United States of America

Erin Raney

United States of America

Manju Rani

India

Geetha Ranmuthugala

Australia

Zeba Rasmussen

United States of America

Jeppe Nørgaard Rasmussen

Denmark

Theo Raynor

United Kingdom

Boika Rechel

United Kingdom

Bernd Rechel

United Kingdom

David Reeves

United Kingdom

Marina Reeves

Australia

Marcus Reidenberg

United States of America

Daniel Reidpath

Malaysia

Sharon Reif

United States of America

Richard Reithinger

United States of America

Sylvia Reitmanova

Canada

Thomas Reynolds

United States of America

Dale Rhoda

United States of America

Raul Ribeiro

United States of America

Henry Rice

United States of America

Suzanne Richards

United Kingdom

Colin Ridyard

United Kingdom

Natalie Riediger

Canada

Arthorn Riewpaiboon

Thailand

Susan Rifkin

United States of America

Donna Rindress

Canada

Jacquie Ripat

Canada

Laetitia Rispel

South Africa

Karen Robb

United Kingdom

Christine Roberts

Australia

Rebecca Roberts

United States of America

Jane Robertson

Australia

Alan Robin

United States of America

Suzanne Robinson

United Kingdom

Bridget Robson

New Zealand

Gary Rodin

Canada

Rashmi Josephine Rodrigues

India

Anne Elizabeth Rogers

United Kingdom

James Rohrer

United States of America

Italia Rolle

United States of America

Ulrich Ronellenfitsch

Germany

Katrina Aminah Ronis

Pakistan

Leslie Roos

Canada

Aldo Rosano

Italy

Barbara Rose

United States of America

Adam Rose

United States of America

Rob Roseby

Australia

Pauline Rosenau

United States of America

Ellen Rosenberg

Canada

Matthew Rosengart

United States of America

Alastair Ross

United Kingdom

Joseph Ross

United States of America

Nan Rothrock

United States of America

Thomas Rotter

Netherlands

David Rowell

Australia

Kevin Rowley

Austria

Renzo Rozzini

Italy

Imre Rurik

Hungary

Dana Rutledge

United States of America

Torleif Ruud

Norway

Lucie Rychetnik

Australia

Joanna Rymaszewska

Poland

Michel Sá

Brazil

Mehdi Saberi-Firooz

Iran

Zia Sadique

United Kingdom

Salim Sadruddin

Canada

Mohsin Saeed Khan

Sweden

Wolfgang Saeger

Germany

Marc Saez

Spain

Najibullah Safi

Afghanistan

Mitsue Saito

Japan

Sanna Salanterä

Finland

Sarah Saleem

Pakistan

Shadi Saleh

Lebanon

Domenico Salvatore

Italy

Neelofar Sami

Pakistan

Bhaven Sampat

United States of America

Miguel San Sebastian

Sweden

Maria-Jose Sanchez-Ruiz

Lebanon

Jane Sandall

United Kingdom

Hogne Sandvik

Norway

Osman Sankoh

Ghana

Sujit Sansgiry

United States of America

Maya Santoro

United States of America

Pantelis Sarafidis

United Kingdom

Pantelis Sarafidis

Greece

Antonio Sarria-Santamera

Spain

Carme Saurina

Spain

Judith Savageau

United States of America

Dennis Scanlon

United States of America

Hendrik Simon Schaaf

South Africa

Ellen Ingrid Schafheutle

United Kingdom

Deborah Schaler

Australia

Joel Schectman

United States of America

Allison Schenk

United States of America

Robert Scherpbier

China

Albert Scherpbier

Netherlands

Stephanie Schim

United States of America

Niklas Schmedt

Germany

Virginia Schmied

Australia

Julie Schmittdiel

United States of America

Eric Schneider

United States of America

Preston Schneider

United States of America

Adrian Schoo

Australia

Beate Schrank

Austria

Jonas Schreyögg

Germany

Matthias Schützwohl

Germany

Sharon Schweikhart

United States of America

Rene Schwendimann

Switzerland

David Sclar

United States of America

Vera Scott

South Africa

Robert Scragg

New Zealand

Federica Secci

United Kingdom

Walter Sermeus

Belgium

Laura Serrant-Green

United Kingdom

Nick Sevdalis

United Kingdom

Omid Shabestari

Canada

Efrat Shadmi

Israel

Asrul Shafie

Malaysia

Parag Shah

United States of America

Sarwat Shah

Pakistan

Shaouli Shahid

Australia

Babar Tasneem Shaikh

Pakistan

P Ravi Shankar

Nepal

Veena Shankaran

United States of America

Christopher Sharpley

Australia

Charles Shaw

United Kingdom

Lisa Shaw

United Kingdom

Wayne Shelton

United States of America

Jing Shen

United Kingdom

Elizabeth Shenkman

United States of America

Geoff Shepherd

United Kingdom

Sasha Shepperd

United Kingdom

Recinda Sherman

United States of America

Sarah Shih

United States of America

Supriya Shore

United States of America

Ronald Shorr

United States of America

Alison Short

Australia

Chris Showell

Australia

Lindiwe Sibeko

Canada

Muhammad Bilal Siddiqui

Pakistan

Kristi Sidney

Sweden

Ali Sie

Burkina Faso

Sheetal Silal

South Africa

Bernard Silke

Ireland

Eva Silvestre

United States of America

Daudi Simba

Tanzania

Steffen T Simon

Germany

Judit Simon

United Kingdom

Alan Simpson

United Kingdom

Hardeep Singh

United States of America

Niroshan Sivathasan

United Kingdom

Diane Skiba

United States of America

Ralf Skripitz

Germany

Kjersti Eeg Skudal

Norway

Adrian Sleigh

Australia

Sandra Small

Canada

Ben J Smith

Australia

Helen J Smith

United Kingdom

Samantha Smith

Ireland

Alesha Smith

Australia

Kim Smolderen

United States of America

Carolyn Snider

Canada

Helen Snooks

United Kingdom

Jeffrey Soar

Australia

Patricia Soarez

Brazil

Shoshanna Sofaer

United States of America

Leif Solberg

United States of America

Nils Gunnar Songstad

Norway

Joann Sorra

United States of America

John Sparrow

United Kingdom

Stuart Speedie

United States of America

JoAnn Sperl-Hillen

United States of America

Allan Spigelman

Australia

Karen Spilsbury

United Kingdom

Matthew Spittal

Australia

Catherine Spooner

Australia

Cor Spreeuwenberg

Netherlands

Peter Spurgeon

United Kingdom

Magi Sque

United Kingdom

Allison Squires

United States of America

Chandrashekhar Sreeramareddy

Nepal

Anthony Staines

Ireland

Cecilia Stålsby Lundborg

Sweden

Hilary Standing

United Kingdom

Cynthia Stanton

United States of America

Tom Stargardt

Germany

Terence Starz

United States of America

Jürgen Stausberg

Germany

Sally Stearns

United States of America

Nicholas Steel

United Kingdom

John Steiner

United States of America

Jost Steinhaeuser

Germany

Karen Stenner

United Kingdom

Anita Stern

Canada

Alistair Stewart

New Zealand

Nigel Stocks

Australia

Björn Stollenwerk

Germany

Roslyn Stone

United States of America

Nicole Stout

United States of America

Bjørn Heine Strand

Norway

Leanne Streja

United States of America

Raymond Strikas

United States of America

Matthew Studenski

United States of America

Therese Stukel

Canada

Tintin Su

Malaysia

Eric Suba

United States of America

Samy Suissa

Canada

Ron Summers

United Kingdom

Shu-Fen Sun

Taiwan

Patricia Sunaert

Belgium

Reijo Sund

Finland

Paibul Suriyawongpaisal

Thailand

Esther Suter

Canada

Kurt Svardsudd

Sweden

Margaret Petrina Sweeney

United Kingdom

Ehsan Syed

United States of America

Sjoerd Sytema

Netherlands

Miriam Taegtmeyer

United Kingdom

Daniel Talmor

United States of America

Michael Tam

Australia

Eleonora Tan

United States of America

Heather Tan

Australia

Toshiaki Tanaka

Japan

Carolyn Tarrant

United Kingdom

Rod Taylor

United Kingdom

David Taylor

United Kingdom

Denise Taylor

New Zealand

Tatiana Taylor

United Kingdom

Gregory Teague

United States of America

Cristian Tebé

Spain

Flora Fang-Hwa Teng

Canada

Tiew-Hwa Katherine Teng

Australia

Alan Tennant

United Kingdom

Laura Ternent

United Kingdom

Marroon Thabane

Canada

Gudrun Theile

Germany

Falk Thiel

Germany

Roger Thomas

Canada

Sarah Thomas

United Kingdom

Sandra Thompson

Australia

Kristina Tiedje

France

Areti Tillou

United States of America

Martin Tobias

New Zealand

Paloma Toledo

United States of America

Stephanie Topp

Zambia

Aleksandra Torbica

Italy

Kwasi Torpey

Nigeria

Anne Townsend

Canada

Huong Tran

United States of America

Marta Trapero-Bertran

Spain

Marta Trapero-Bertran

United Kingdom

Catherine Travers

Australia

Katherine Treiman

United States of America

Paolo Trerotoli

Italy

Shaun Treweek

United Kingdom

Chetan Trivedy

United Kingdom

Tsuen-Chiuan Tsai

Taiwan

Komla Tsey

Australia

Miyuki Tsuchihashi-Makaya

Japan

Philip Tucker

United Kingdom

Janet Turan

United States of America

Tari Turner

Australia

Aage Tverdal

Norway

Liina-Kaisa Tynkkynen

Finland

Audrey Umbreit

United States of America

Carolyn Unsworth

Australia

Christine Urquhart

United Kingdom

Leana Uys

South Africa

Benjamin S. Chudi Uzochukwu

Nigeria

Hannu Valtonen

Finland

Sara Van Belle

Belgium

Wim Van Damme

Belgium

Joris van de Klundert

Netherlands

Hendrik van den Bussche

Germany

Koen Van den Heede

Belgium

Jan Van der Meulen

United Kingdom

Philip Van der Wees

Netherlands

Nynke Van Dijk

Netherlands

Arna van Doorn - Klomberg

Netherlands

Sandra van Dulmen

Netherlands

Courtney Van Houtven

United States of America

Charles Van Liew

United States of America

Gijsberta van Londen

United States of America

Carl van Walraven

Canada

Jeroen van Wijngaarden

Netherlands

Brian van Wyk

South Africa

Marta van Zanten

United States of America

Per Olav Vandvik

Norway

Inge Varekamp

Netherlands

Emma Varley

Canada

Allison Vaughn

United States of America

David Vawdrey

United States of America

Isabelle Vedel

Canada

Marijke Veenstra

Norway

Ketan Vegda

Canada

Andrew Veitch

United Kingdom

Elizabeth Venditti

United States of America

Else Vengnes Grue

Norway

Hilde Verbeek

Netherlands

Katia Verhamme

Netherlands

Robert A Verheij

Netherlands

Marja Verhoef

Canada

Dante Verme

United States of America

Michael Verta

United States of America

Joshua Vest

United States of America

Michel Vezina

Canada

Catherine Vialle-Valentin

United States of America

James Vickers

Australia

Victor Vickland

Australia

Edgar Vieira

United States of America

Rebecca Vigen

United States of America

Irene Vikman

Sweden

Stefano Villa

Italy

Tereza Villa

Brazil

Elmer Villanueva

Australia

Philip Vita

Australia

Agnes Vitry

Australia

Olaf von dem Knesebeck

Germany

Hubertus JM Vrijhoef

Netherlands

Hubertus Vrijhoef

Singapore

Cordula Wagner

Netherlands

Brittin Wagner

United States of America

Rolf Wahlstrom

Sweden

Bonnie Wakefield

United States of America

John Wakerman

Australia

Nicholas Waldron

Australia

Kylie Wales

Australia

David Walker

Canada

Bruce Walker

Australia

Lorraine Wallace

United States of America

Kieran Walshe

United Kingdom

Scott Walter

Australia

Fiona Walter

United Kingdom

Gilles Wandeler

Switzerland

Jianming Wang

China

Wei Wang

Australia

Lu Wang

Canada

Jeanette Ward

Australia

Justin Waring

United Kingdom

Nicole Warren

United States of America

Jason Washburn

United States of America

Graham Watt

United Kingdom

Cynthia Webster

Australia

Robert Weech-Maldonado

United States of America

Tarun Weeramanthri

Australia

Ralf Weigel

United Kingdom

Yvonne Wells

Australia

Kuang-Yi Wen

United States of America

Michel Wensing

Netherlands

Alan West

United States of America

Gert P. Westert

Netherlands

Kath Weston

Australia

Randy Wexler

United States of America

Simone Weyers

Germany

Victoria White

Australia

Dean Whitehead

New Zealand

Kirsten Whitehead

United Kingdom

Nedra Whitehead

United States of America

ANdrea Whittaker

Australia

Birgitt Wiese

Germany

John Wiggers

Australia

Torsten Wilhelm

Germany

Lesley Wilkes

Australia

Lynsey Willenberg

Australia

Anna-leila Williams

United States of America

Allison Williams

Canada

Gareth Williams

United Kingdom

Rhys Williams

United Kingdom

Don Willison

Canada

Sarah Wilson

Canada

Marilyn Wise

Australia

Meg Wise

United States of America

Marilyn Wise

Australia

Jennifer Wittwer

United States of America

Fredric Wolinsky

United States of America

Brian Wong

Canada

Geoff Wong

United Kingdom

Robert Woodward

United States of America

Tanja Wörmann

Germany

Emiel Frans Maria Wouters

Netherlands

Linda Wray

United States of America

Yung-Hung Wu

Taiwan

Chen-Yi Wu

Taiwan

Gwenllian Wynne-Jones

United Kingdom

Kaspar Wyss

Switzerland

Imam Xierali

United States of America

Yantao Xin

China

Fujie Xu

United States of America

Chuangzhou Xu

China

Andreas Xyrichis

United Kingdom

Atsuko Yamaguchi

Japan

Chiang-Hsing Yang

Taiwan

Ming-Chin Yang

Taiwan

Nan-Ping Yang

Taiwan

Robert Yates

Indonesia

Maurice Yé

Burkina Faso

Jane Yelland

Australia

Laurann Yen

Australia

Veerasamy Yengopal

South Africa

P. Stanley Yoder

United States of America

Jean Yoon

United States of America

Lance Brendan Young

United States of America

M. Scott Young

United States of America

Doris Young

Australia

Sitaporn Youngkong

Thailand

Yunxian Yu

China

Shahaduz Zaman

United Kingdom

Khalequz Zaman

United States of America

Leonidas Zampetakis

Greece

Margareth Zanchetta

Canada

Ehsan Zarei

Iran

Tija Zarkovic Palijan

Croatia

Thomas Zastowny

United States of America

John Zeber

United States of America

Peter Zed

Canada

Jennifer Zelmer

Canada

Chunliu Zhan

United States of America

Xuanping Zhang

United States of America

Yimin Zhang

China

Yuejen Zhao

Australia

Liebin Zhao

China

Xiaoying Zheng

China

Chao Zhou

United States of America

Joseph Zickafoose

United States of America

Jeanette Ziegenfuss

United States of America

Charlotte Muheki Zikusooka

Uganda

David Zimmer

United States of America

Marloes Zuidgeest

Netherlands

Pascal Zurn

Switzerland

## Pre-publication history

The pre-publication history for this paper can be accessed here:

http://www.biomedcentral.com/1472-6963/13/78/prepub

